# Sexual health after childbirth in Dutch women: prevalence, associated factors and perceived need for information: a cross-sectional study

**DOI:** 10.1186/s12884-024-06918-w

**Published:** 2024-12-20

**Authors:** EL Wassenaar, F Lont, CJ Verhoeven, J Henrichs, LML Titulaer, JC Warmelink, CC Geerts

**Affiliations:** 1 Midwifery Academy Amsterdam Groningen, Inholland, Amsterdam, the Netherlands; 2 Midwifery Academy Amsterdam Groningen, Inholland, Groningen, the Netherlands; 3 Amsterdam UMC location Vrije Universiteit Amsterdam, Midwifery Science, De Boelelaan 1117, (1081 HV), Amsterdam, the Netherlands; 4 Amsterdam Public Health, Quality of Care, Amsterdam, the Netherlands; 5 Division of Midwifery, School of Health Sciences, University of Nottingham, Nottingham, UK; 6 Department of Obstetrics and Gynaecology, Maxima Medical Centre, Veldhoven, the Netherlands; 7 Department of Primary and Long-term Care, University of Groningen, University Medical Center Groningen, PO Box 196, Groningen, 9700 AD the Netherlands

**Keywords:** Sexual distress, Sexual dysfunction, Birth experience, Associated factors, Sexual health, Childbirth, Postpartum

## Abstract

**Background:**

After childbirth, women often experience changes in sexual health. Little is known about the associated factors for the development of sexual health problems. Therefore, in this study we aim to investigate (1) how many women report changes in sexual health; (2) the prevalence and associated factors of women’s postpartum sexual health problems and (3) whether women want to be better informed about postpartum sexual health after childbirth.

**Methods:**

From March to May 2021, 641 postpartum Dutch women participated in a cross-sectional study through an online survey, distributed via social media. At a mean time of 11.6 months (SD = 6.23) after birth, women reported information on maternal, pregnancy and childbirth characteristics and childbirth experience, sexual dysfunction (Female Sexual Function Index), sexual distress (Female Sexual Distress Scale), relationship satisfaction (Relationship Assessment Scale) and “perceived need for information” to discuss sexual health with their maternity care provider.

**Results:**

Postpartum changes in sexual health were found in 88% of women. Of the women who participated, 43.7% reported sexual dysfunction and 52.3% reported sexual distress. Overall, 46% of women perceived more need for information. Multivariable logistic regression analyses showed that negative sexual experiences were associated with increased odds of sexual dysfunction (odds ratio (OR) 1.58, 95% CI 1.04–2.40) and sexual distress (OR 1.70, 1.17–2.46). Perineal damage (OR 1.54, 1.03–2.29) was associated with increased odds of sexual dysfunction, and a BMI ≥ 30 kg/m^2^ (OR 0.46, 0.28–0.70) was associated with decreased odds of sexual dysfunction. A higher level of relationship satisfaction was associated with decreased odds of sexual dysfunction (OR 0.36, 0.25–0.51) and sexual distress (OR 0.47, 0.35–0.63). A positive childbirth experience was associated with decreased odds of sexual distress (OR 0.88, 0.81–0.96) and decreased odds of “perceived need for information” (OR 0.86, 0.79 to 0.94).

**Conclusion:**

Sexual health problems are experienced by half of postpartum women. Midwives should inform women and their partners about these possible problems, taking into account birth-related factors, e.g. birth experience and perineal damage. Care providers should pay special attention to women with negative sexual experiences.

**Supplementary Information:**

The online version contains supplementary material available at 10.1186/s12884-024-06918-w.

## Background

 Sexual health can be described as physical, emotional, mental and social wellbeing in respect to sexuality and contributes to general wellbeing and (mental) health [1]‎. Life-events, physical or psychological conditions, and various medical interventions can affect sexual health [2]. Poor sexual health affects not only women’s general wellbeing and health but also the quality of their partner relationship [3–5]. Moreover, sexual health problems negatively affect women’s quality of life [6].

An Australian cross-sectional study of 489 postpartum women showed that 64.3% experienced sexual dysfunction in the first year postpartum [5]. Among those experiencing sexual dysfunction, the most common problems included disruption of sexual desire (81.2%), orgasmic problems (53.4%), and loss of sexual arousal (52.3%)^5^. A longitudinal cohort study among 832 primiparous women demonstrated that approximately half of these women had self-perceived sexual health problems at six months postpartum [7]. In this study, the most prevalent sexual problems included loss of interest in sexual activity up to one year after giving birth (39.8%) and pain during intercourse (dyspareunia) six months postpartum (37.5%) [7]. Until now, the prevalence of sexual health problems among Dutch postpartum women has been unknown.

Studies suggest that several medical and postpartum psychosocial factors are related to the development of postpartum sexual problems. Khajehei et al. [5] reported that primiparity and reduced sexual activity, depression and relationship dissatisfaction in the postpartum period are associated with sexual health problems. Breastfeeding is associated with vaginal dryness, dyspareunia and loss of interest in sexual activity [7]. Third-degree tears are related to dyspareunia [7, 8]. The main reasons women reported for not having continued sexual intercourse were not being interested, being too tired, fearing pain, dyspareunia and decreased vaginal lubrication [9]. Some studies report no direct link between mode of birth and perineal trauma and sexual health [8, 10, 11], however a recent systematic review [12] reported that sexual function can be affected by mode of birth, especially assisted vaginal birth was associated with worse sexual function. More research into associated factors for postpartum sexual health problems is needed as this can offer opportunities to improve the early identification of these problems and to develop and/or optimise timely preventative interventions addressing this matter.

Although sexual health is an important component of both somatic and mental health, maternity care providers currently pay little attention to the subject of sexual health. A Canadian study reported that 76% of pregnant women, for whom sexual activity was not discussed during pregnancy, felt the need to address this topic [13]. The “Basic Prenatal Care” guidelines of the Dutch Association of Obstetrics and Gynaecology (NVOG) [14] and the Royal Dutch Organisation of Midwives (KNOV) standard “Prenatal Midwifery Care” [15] do not address this subject. The multidisciplinary guideline “Postnatal Care” mentions that resumption of sexual intercourse can be discussed during birth control counseling six weeks postpartum [16]. However, discussing postpartum sexual health during or after pregnancy is not routine care, and it is unknown how often this is actually discussed in the Netherlands. Therefore, research into Dutch women’s “perceived need for information” concerning sexual health in the pre- and postpartum periods should be conducted.

The general aim of the current survey is to gain insight into the prevalence of sexual health problems and “perceived need for information” of Dutch postpartum women and to explore the association between maternal and obstetric factors and sexual health problems and “perceived need for information”, separately. First, in this study, the prevalence of sexual health problems in Dutch postpartum women is evaluated. Since sexuality is strongly influenced by many sociocultural factors, such as religion, expectations and believes [2], it is not known whether the prevalence of sexual health problems is comparable across countries. The Netherlands has favourable sexual health outcomes [17], which could hypothetically lead to a lower prevalence of sexual health problems among Dutch postpartum women. However, this prevalence is currently unknown. Second, this study investigated which demographic, peripartum, obstetric and psychosocial determinants are associated with sexual health problems postpartum among Dutch women. Third, we assessed how often sexual health was discussed by maternity care providers and how many postpartum women wanted to be informed about sexual health (“perceived need for information”). To gain insight into the characteristics of women who perceived (more) “need for information”, we also investigated determinants of “perceived need for information”, to inform maternity care providers about how to provide more tailored care.

## Methods

### Study design and study population

For the current study we conducted a cross-sectional survey among Dutch postpartum women. Participants were recruited between February 2021 and May 2021. Data were collected via an online questionnaire, developed in Survalyzer Next Generation (https://survalyzer.com). Women aged 18 years and older, who had given birth in the last two years and were able to read and understand the Dutch language were eligible to participate. Women were included if they had a vaginal birth or a caesarean section. Women who were pregnant at the time of completing the questionnaire were excluded. The link to the online questionnaire was shared with participants on social media platforms (such as Facebook, Instagram, Twitter and LinkedIn). All participants were informed about the study via an introduction on the first page of the online questionnaire and provided digital informed consent before accessing the online questionnaire.

### Ethical approval

This study was approved by the Medical Ethics Committee (METC) of Amsterdam University Medical Centre (2020.0744, 3/2/2021).

## Measures

### Sexual dysfunction and sexual distress

To measure sexual health problems, the Female Sexual Function Index (FSFI) [18] and the Female Sexual Distress Scale (FSDS) [19] were used. These self-report questionnaires have been validated in a Dutch setting [20, 21] and measure female sexual function (FSFI) and female sexually related personal distress (FSDS).

The FSFI assesses sexual function in the past four weeks and contains 19 questions that can be answered on a Likert scale, with scores ranging from 1 to 5 (‘1 = almost never or never’; ‘5 = almost always or always’) or from 0 to 5 (0 indicating no sexual activity). The questions address six domains: desire, arousal, lubrication, orgasm, satisfaction and pain. Following the instructions of ter Kuile et al. [21] each domain was multiplied by a value ranging from 0.3 to 0.6 [21]. The total score range from 2 to 36, with lower scores indicating lower levels of sexual functioning. The variable was dichotomized, with a total score of 26.55 or less is defined as female sexual dysfunction [22]. The sensitivity of the FSFI in a Dutch population is 96,7% and the specificity is 88,0% for detecting sexual dysfunction [21]. Of the 19 FSFI items, 15 items have a response option of “no sexual activity” or “did not attempt intercourse”, which is assigned a score of zero if selected. In line with the recommendations of Meyer-Bahlburg & Dolezal 2007 women who indicated no sexual activity/ intercourse in the last 30 days were excluded from the analysis, as these women were most likely not sufficiently sexually active for the FSFI to be a valid assessment of their sexual functioning [23, 24]. The Cronbach’s alpha for the FSFI in our study was 0.97.

The FSDS comprises 12 questions concerning aspects of sexual distress in women, e.g. “How often have you felt sad about your sex life?” The sensitivity in a Dutch population is 92.2% and the specificity for detecting sexual distress is 76.6% [21]. Each question can be scored on a 5-point Likert scale (0 = never to 4 = always). The total sum score ranges from 0 to 48 [21]. The variable was dichotomized, with a total score of 15 or more indicating sexual distress [21]. The Cronbach’s alpha for FSDS in our study was 0.95.

To gain insight into whether women experience changes in sexual health after birth, the following questions were asked: “Did you experience changes in sexual health postpartum?” (yes/ no) and “If yes, what were these changes?” (Sexual activity is less frequent; I experience less interest in sexual activity; I find it harder to become sexually aroused; Sexual activity is painful; It is difficult to become lubricated (“wet”); It is harder to reach an orgasm/orgasm is less frequent; or Other changes, namely…).

### Determinants

Using the online questionnaire women were asked to provide information on demographics, obstetric and other medical factors, and psychosocial factors to obtain information on potential determinants of postpartum sexual health problems.

#### Maternal characteristics

 Women reported parity (primiparous, multiparous), maternal age in years at the time of completing the questionnaire, which was divided into three categories (< 30, 30–34, ≥ 35 years), and gestational age at birth, which was divided into three categories (< 37, 37–40, ≥41 weeks). Ethnic background was based on the definition used by Statistics Netherlands (2020), which considers someone to be of non-Dutch ethnicity if at least one of the parents was born in a country other than the Netherlands. A distinction was made between Dutch ethnicity, another Western ethnicity (Europe (all countries except Turkey), North America and Oceania, Indonesia and Japan) and non-Western ethnicity (all other countires) [25]. Educational level was defined, depending on the highest level of achievement, as low (low to medium-level secondary education or first half of higher-level secondary education), medium (higher-level secondary education or vocational education) or high (university of applied sciences or university education and higher) [26]. Body Mass Index (BMI) at the time of questionnaire completion was calculated based on length and weight of the participant and categorised as underweight (< 18.5 kg/m^2^), healthy weight (18.5–24.9 kg/m^2^), overweight (25.0–29.9 kg/m^2^) or obese (≥ 30.0 kg/m^2^) [27]. Having had a negative sexual experience in the past (yes or no) was also measured. The time of assessment, when the questionnaire was completed after birth, was expressed in months and categorised into: 0–5 months, 6–11 months, 12–18 months or more than 18 months.

#### Obstetric and medical factors

 Mode of conception was dichotomized as spontaneous or nonspontaneous, including ovulation induction, insemination, intrauterine insemination, in vitro fertilisation or intracytoplasmic sperm injection. Mode of birth was categorised as spontaneous vaginal birth, assisted vaginal birth or caesarean section. Episiotomy was reported as yes or no, and perineal trauma that needed stitching was reported as yes or no. Breastfeeding was assessed and categorised as follows: no; or yes: 0 to 3 months (first week, six weeks or 3 months in the questionnaire), 3 to 6 months, or more than 6 months.

#### Birth experience, psychological factors and relationship assessment

 Birth experience was rated on a scale from 1, indicating a very negative birth experience, to 10, indicating a very good birth experience. Current psychological complaints for which women consulted a psychologist/psychiatrist (yes or no) were assessed. The Relationship Assessment Scale (RAS) was used to assess relationship satisfaction [28]. The Relationship Assessment Scale is a self-report measure of global relationship satisfaction [28]. It consists of seven items, each rated on a five-point Likert scale. Examples of answer options are ‘1 = Poorly’ to ‘5 = Extremely well’. It is suitable for use with individuals who are in intimate relationships. The score can range from 7 to 35 with a high score indicating a high level of satisfaction [28]. The Cronbach’s alpha for the RAS in our study was 0.89.

### Perceived need for information

The following questions were constructed to assess the way and kind of sexual health information was provided to postpartum women by maternity care providers in the Netherlands. The question “Did your maternity care provider inform you about possible changes in sexuality postpartum?” was asked to gain insight into the current postpartum care around sexual health in the Netherlands; the answer options were: “yes” or “no”. The perceived need for information was assessed by the question “Did you feel the need to get (more) information about the changes in sexuality postpartum?” This variable will be referred to as “perceived need for information”. Women who answered “yes” were considered to have “perceived need for information”. The following question was asked: “When did you need this information most?” The answer options were: “In the first trimester”, In the second trimester”, “In the third trimester”, “In the first week postpartum”, “At the 6-week postpartum maternity care consult”, “6 weeks to 3 months after childbirth”, “3 to 6 months after childbirth” and “More than 6 months after childbirth”. Furthermore, the question: “How often should changes in sexuality be discussed during pregnancy and/or the postpartum period?”, was asked, with answer options ranging from 0 to 6 or more. The question “Who, in your opinion, is the best person to discuss sexuality with postpartum?” could be answered by “Midwife”, “Obstetrician”, “Maternity Care nurse” (Table 1), “Lactation consultant”, “General Practitioner” and “Other women/mothers”. The last question was “Why did you choose this person?” which could be answered as follows: “I saw this person most during pregnancy”, “This person feels the most familiar”, “This person knows the most about sexuality” and “Other reason”.

**Table 1:** Maternity care nurse or “kraamzorg”

In the Netherlands midwives are responsible for the care of women with low risk pregnancies and births. In the case of high risk pregnancies clinical midwives, doctors and obstetricians are responsible for care. After giving birth in a hospital in the Netherlands, women go home within a few hours if no complications have occurred, or they are already at home in case of a home-birth. During the first week a “maternity care nurse” will visit them at home for 3-8 hours a day to help them take care of the baby and perform routine health measures. A midwife will make several home visits during the first week after birth to assess the mothers’ and babies’ health. The maternity care nurse reports back to the midwife if problems arise during the postpartum period.	

## Data Analysis

### Sample size

Based on previous work [5, 7] we conservatively assumed a prevalence of postpartum sexual dysfunction of 35%. A theoretical sample of 500 women would have 175 cases with postpartum sexual dysfunction. Such a sample size would thus allow to include 17 determinants in a logistic regression model when taking into account the 10 cases per determinant rule into account [29]. Therefore, we aimed to include at least 500 women for the current study. 

### Prevalence

The FSFI and FSDS total scores were categorised into dichotomous outcomes (see measures) [20]. “Perceived need for information” concerning sexual health was described as “feeling the need to get (more) information about the changes in sexual health postpartum”. The prevalence of sexual dysfunction (FSFI total score ≤ 26.55) and sexual distress (FSDS total score ≥ 15) and nature of the changes in sexuality experienced by women after childbirth is presented as frequencies and percentages using descriptive statistics.

### Associated factors sexual dysfunction, sexual distress and perceived need for information

A univariable logistic regression analysis was used with sexual dysfunction and distress and “perceived need for information” as the dependent variables to analyse which maternal, demographic and intrapartum factors were associated with sexual health problems or “perceived need for information”. Crude odds ratios (ORs) with 95% confidence interval (CIs) were reported.

The variables were subjected to a multivariable hierarchical logistic regression analysis on the basis of a conceptual hierarchical framework [30]. According to this framework, maternal characteristics (parity, age, gestational age, ethnicity, education level, BMI, negative sexual experience) were entered first into the analysis. Obstetric and other medical factors (mode of conception, mode of birth, episiotomy, perineal trauma, breastfeeding) were added in the second step. In the third step, birth experience, psychological factors and RAS were entered into the model. To be able to identify all relevant determinants, the cut-off for the p-value in this analysis was set higher than traditional levels, that is, a p-value < 0.15 [31]. So, in each step, all the variables from that category were entered into the model, and only variables with a p-value < 0.15 were kept in the following step. Thus, in the fourth step, a final model was built that included only variables with a p-value < 0.15 from the previous steps. The adjusted odds ratios (adjORs) and 95% CIs from the final model were reported. Determinants with a p-value < 0.05 in the final model were considered to be statistically significantly associated with sexual dysfunction and distress and “perceived need for information”. For individual associated factors with at least 5% and less than 30% missing data, a multiple imputation procedure applying 20 imputed sets was carried out, which was based on the calculated predictive distribution derived from the associations between all associated factors, to replace missing values. In this research, all odds ratios represent pooled effect estimates based on the 20 imputed sets. All analyses were performed using SPSS Statistics version 26.

## Results

### Study Population

**Fig. 1:**
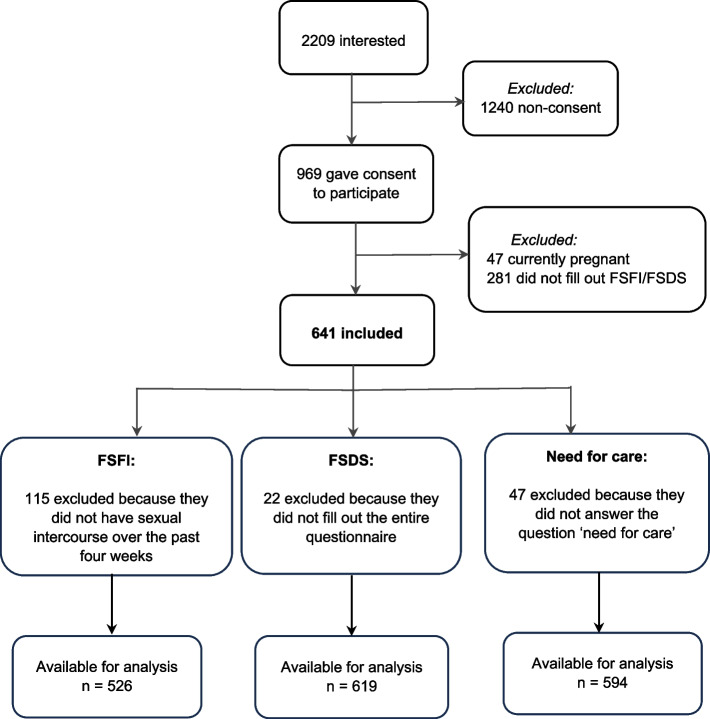
Flowchart of the study sample

Figure 1 shows that 2209 people were interested in the study, of whom 969 gave consent to participate. Among these 969 women, 328 were excluded because they did not meet the criteria or did not complete the FSFI or FSDS. A total of 641 women were included in one or more of the main analyses of the current study. Data for the FSFI were available for 526 women; for 115 women, this instrument was not applicable because they reported not having sexual intercourse in the four weeks before completing the questionnaire, and these women were excluded from the analysis. The FSDS questionnaire data were available for 619 women; 22 did not fill out this questionnaire but did fill out the FSFI, 594 women filled out the questions about “perceived need for information”

The total study sample (*n* = 641) included 354 (55.2%) primiparous women and 287 (44.8%) multiparous women. The ages ranged from 19 to 42 years. Among the study population 85.3% (*n* = 547) was Dutch, 10.3% (*n* = 66) had a Western background and 4.2% (*n* = 27) had a non-Western background. Among the study sample, 8.4% (*n* = 54) had a low education level, 35.1% (*n* = 225) had a medium education level and 56.5% (*n* = 362) were highly educated. These and other characteristics are shown in Table 2.

**Table 2:** Univariable logistic regression analysis of characteristics and sexual dysfunction (FSFI ≤26,55) and sexual distress (FSDS ≥15)

	**Total**	**Sexual Dysfunction**	**Crude OR ** **(95% CI)**	**Sexual Distress**	**Crude OR ** **(95% CI)**
	*n* = 641	230/526 (43.7%)		324/619 (52.3%)	
**Maternal characteristics**					
**Parity**					
Primiparous	354 (55.2)	135/294 (45.9)	reference	173/340 (50.9)	reference
Multiparous	287 (44.8)	95/232 (40.9)	0.82 (0.58-1.16)	151/279 (54.1)	1.14 (0.83-1.56)
**Maternal age**					
<30	256 (39.9)	105/216 (48.6)	1.31 (0.90-1.91)	130/245 (53.1)	0.99 (0.70-1.41)
30-34	263 (41.0)	91/217 (41.9)	reference	136/255 (53.3)	reference
≥35	122 (19.0)	34/93 (36.6)	0.80 (0.48-1.32)	58/119 (48.7)	0.83 (0.54-1.29)
**Gestational age**					
<37 weeks	30 (4.7)	11/26 (42.3)	0.91 (0.40-2.03)	14/30 (46.7)	0.78 (0.37-1.63)
37-40 weeks	484 (75.5)	176/394 (44.7)	reference	246/464 (53.0)	reference
≥41 weeks	127 (19.8)	43/106 (40.6)	0.85 (0.55-1.31)	64/125 (51.2)	0.93 (0.63-1.38)
**Ethnic background**					
Dutch	547 (85.5)	199/458 (43.4)	reference	276/530 (52.1)	reference
Western	66(10.3)	22/50 (44.0)	1.02 (0.57-1.84)	31/61 (50.8)	0.95 (0.56-1.62)
Non-Western	27 (4.2)	9/17 (52.9)	1.46 (0.55-3.86)	17/27 (63.0)	1.56 (0.70-3.48)
Missing	1				
**Education level**					
Low	54 (8.4)	15/41 (36.6)	0.75 (0.38-1.47)	27/51 (52.9)	1.07 (0.59-1.93)
Middle	225 (35.1)	85/187 (45.5)	1.08 (0.75-1.56)	116/215 (54.0)	1.11 (0.79-1.56)
High	362 (56.5)	30/298 (43.6)	reference	181/353 (51.3)	reference
**Body Mass Index** (imputed)*					
<18.5 kg/m^2^	14 (2.2)	7/12 (58.3%)	-	7/13 (53.4)	-
18.5-24.9 kg/m^2^	265 (41.7)	108/222 (48.6)	reference	138/256 (53.9)	reference
25-29.9 kg/m^2^	204 (32.1)	71/164 (43.3)	0.81 (0.53-1.24)	108/197 (54.8)	1.03 (0.69-1.52)
≥30 kg/m^2^	153 (24.0)	42/124 (33.9)	**0.54 (0.34-0.86) **	69/147 (46.9)	0.76 (0.50-1.15)
**Negative sexual experience**					
Yes	191 (29.8)	75/144 (52.1)	**1.60 (1.08- 2.34)**	118/185 (63.8)	**1.95 (1.37-2.78)**
No	450 (70.2)	155/382 (40.6)	reference	206/434 (47.5)	reference
**Postpartum at **					
0-5 months	100 (15.7)	35/71 (49.3)	reference	47/96 (49.0)	reference
6-11 months	242 (37.9)	92/203 (45.3)	0.85 (0.50-1.47)	130/232 (56.0)	1.33 (0.83-2.14)
12-17 months	165 (25.9)	57/138 (41.3)	0.72 (0.41-1.29)	76/160 (47.5)	0.94 (0.57-1.57)
18+ months	131 (20.5)	45/111 (40.5)	0.70 (0.39-1.28)	69/128 (53.9)	1.22 (0.72-2.07)
Missing	3				
**Obstetric and medical factors**					
**Mode of conception**					
Spontaneous	584 (91.4)	215/484 (44.4)	reference	296/563 (52.6)	reference
Non-spontaneous	55 (8.6)	15/41 (36.6)	0.72 (0.37-1.40)	27/54 (50.0)	0.90 (0.52-1.58)
Missing	2				
**Mode of birth**					
Spontaneous vaginal	502 (78.3)	186/410 (45.4)	reference	260/484 (53.7)	reference
Assisted vaginal birth	55 (8.6)	18/47 (38.3)	0.75 (0.40-1.39)	27/54 (50.0)	0.86 (0.49-1.51)
Caesarean Section	84(13.1)	26/69 (37.7)	0.73 (0.43-1.23)	37/81 (45.7)	0.72 (0.45-1.16)
**Episiotomy **					
Yes	128 (20.0)	44/106 (41.5)	0.89 (0.58-1.38)	71/126 (56.3)	1.23 (0.83-1.82)
No	513 (80.0)	186/420 (44.3%)	reference	253/493 (51.3)	reference
**Perineal trauma that needed stitching**					
Yes	194 (30.3)	82/158 (51.9)	**1.60 (1.10-2.33)**	101/186 (54.3)	1.12 (0.79-1.58)
No	447 (69.7)	148/368 (40.2)	reference	223/433 (51.5)	reference
**Breastfeeding**					
No	127 (19.8)	41/110 (37.3)	reference	61/120 (50.8)	reference
Yes, 0-3 months	163 (25.4)	60/139 (43.2)	1.28 (0.77-2.13)	81/156 (51.9)	1.05 (0.65-1.68)
Yes, 3-6 months	69 (10.8)	21/53 (39.6)	1.10 (0.56-2.16)	32/67 (47.8)	0.88 (0.49-1.61)
Yes, more than 6 months	282 (44.0)	109/224 (48.2)	1.57 (0.98-2.50)	150/276 (54.3)	1.15 (0.75-1.77)
**Birth experience, psychological factors and relationship assessment**					
**Birth experience score from 1 to 10**, mean (SD)	7.51 (2.06)	-	0.95 (0.87-1.03)	-	**0.87 (0.80-0.94)**
**Psychological complaints**					
Yes	71 (11.1)	28/59 (47.5)	1.19 (0.69-2.04)	44/69 (63.8%)	**1.70 (1.01-2.85) **
No	570 (88.9)	202/467 (43.3)	reference	280/550 (50.9%)	reference
Relationship assessment scale from 1 to 7**, mean (SD)	4.22 (0.60)	-	**0.37 (0.27-0.53)**	-	**0.45 (0.33-0.60) **

All values are numbers and percentages; n (%), unless otherwise indicated. Bold values indicate *p*-values < 0.05. * BMI 14.7% missing (*n*=94); ** filled in by n=629 with partners; n=12 had no partner

Among the 526 women who provided valid data on the FSFI, 230 (43.7%) scored ≤ 26.55, indicating sexual dysfunction. Of the 619 women that filled out the FSDS 324 (52.3%) women scored ≥ 15, indicating sexual distress. In addition, 46.0% of the women (273 out of 594 respondents) indicated a “perceived need for information”.

**Fig. 2:**
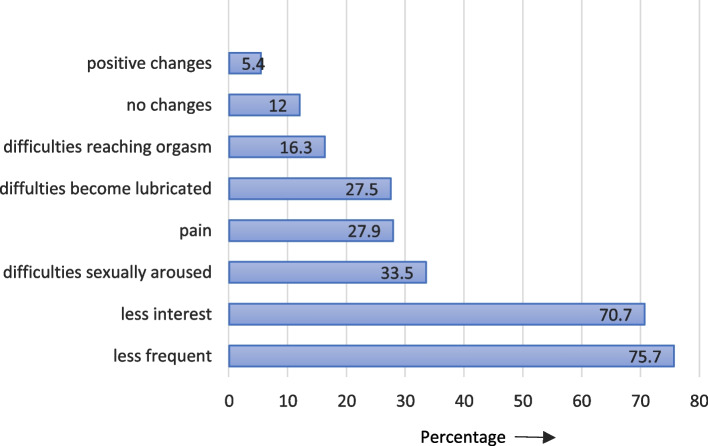
Most reported sexual changes in the postpartum period

Postpartum changes in sexual health were self-reported by 88% (523/594) of women (Figure 2). The changes reported most frequently were less frequent sexual activity (75.7%) and less interest in sexual activity (70.7%). Other women experienced difficulties in becoming sexually aroused (33.5%), pain during sexual activity (27.9%) or difficulties in becoming lubricated (27.5%). Some women (16.3%) had difficulties reaching an orgasm or orgasmed less frequently. A small group of women reported positive changes (5.4%), including increased libido and becoming lubricated more easily and more easily reaching an orgasm.

### Associated factors for sexual dysfunction and sexual distress

Table 2 shows the results of the univariable logistic regression analyses of the maternal, obstetric and medical determinants and birth experience, psychosocial factors and RAS of postpartum sexual dysfunction and sexual distress.

A negative sexual experience was associated with increased odds of sexual dysfunction and sexual distress (OR 1.60, 95% CI 1.08–2.34 and OR 1.95, 95% CI 1.37–2.78, respectively), whereas positive relationship satisfaction (RAS) was related to a decreased odds of sexual dysfunction and sexual distress (OR 0.37 95% CI 0.27–0.53; OR 0.45, CI 0.33–0.60, respectively). Perineal trauma was associated with increased odds of sexual dysfunction (OR 1.60, 95% CI 1.10–2.33), and BMI ≥ 30 kg/m^2^ was associated with a decreased odds of sexual dysfunction (OR 0.54 95% CI 0.34–0.86). Psychological complaints were associated with increased odds of sexual distress (OR 1.70, 95% CI 1.01–2.85). A positive birth experience was, however, related to a decreased odds of sexual distress (OR 0.87, 95% CI 0.80–0.94).

The results of the multivariable hierarchical logistic regression model investigating the associated variables with sexual dysfunction and sexual distress, respectively, are shown in Table 3. Associated factors with a *p*-value < 0.15 were included in this model. The previous steps of the model building, reporting the *p*-values of the characteristics in each step, are shown in supplementary file 1.

**Table 3:** Multivariable logistic regression analysis of sexual dysfunction (FSFI ≤26,55) and sexual distress (FSDS ≥15)

	**Adjusted OR** **(95% CI)** **Sexual Dysfunction**	***p-value***	**Adjusted OR** **(95% CI)** **Sexual Distress**	***p-value***
**Maternal characteristics**				
**Body Mass Index (imputed)**				
<18.5 kg/m^2^	1.07 (0.30-3.86)	0.92		
18.5-24.9 kg/m^2^	reference	-		
25-29.9 kg/m^2^	0.76 (0.49-1.12)	0.24		
≥30 kg/m^2^	0.46 (0.28-0.75)	**0.002**		
**Negative sexual experience**				
Yes	1.58 (1.04-2.40)	**0.03**	1.70 (1.17-2.46)	**0.01**
No	reference		reference	
**Obstetric and medical factors**				
**Perineal trauma that needed stitching**				
Yes	1.54 (1.03 – 2.29)	**0.04**		
No	reference			
**Birth experience, psychological factors and relationship assessment**				
** Birth experience Score from 1 to 10**			0.88 (0.81-0.96)	**0.003**
** Relationship assessment scale**	0.36 (0.25-0.51)	**< 0.001**	0.47 (0.35-0.63)	**<0.001**

Perineal trauma requiring stitching was associated with sexual dysfunction (OR 1.54 95% CI 1.03–2.29). Negative sexual experience was associated with sexual dysfunction and sexual distress (OR 1.58, 95% CI 1.04–2.40 and OR 1.70, 95% CI 1.17–2.4, respectively). Higher levels of relationship satisfaction (RAS) were, however, associated with lower odds of sexual dysfunction and sexual distress (OR 0.36 95% CI 0.25–0.51; and OR 0.47, CI 0.35–0.63, respectively). BMI ≥ 30 kg/m2 was associated with a lower likelihood of sexual dysfunction (OR 0.46 95% CI 0.28–0.75) and more positive birth experiences were associated with lower likelihood of sexual distress (OR 0.88, 95% CI 0.81–0.96).

### Perceived need for information

Among all the women who completed the “perceived need for information”-questionnaire (*n* = 594) 75.8% (*n* = 450) reported that they did not receive information about possible changes in sexuality postpartum from their midwives or other healthcare providers. Additionally, 46.0% (*n* = 273) of women felt the need to have (more) information about sexual health postpartum. Most women wanted to receive this information at the 6-week check-up (29.0%, *n* = 172). Others wanted to receive this information between 6 weeks and 3 months postpartum (26.6%, *n* = 158). For most women, discussing this topic once (39.6%) or twice (32.0%) was enough. The midwives were perceived as the best care providers for giving this information according to 488 women (82.2%), although 29.8% (*n* = 177) reported it to be the “maternity care nurse” and 24.2% (*n* = 144) reported it should be the obstetrician. The reasons for choosing a certain care provider to discuss sexual health, were mainly to feel most familiar (60.4%) with that care provider and to have the most frequent contact with him or her during pregnancy (53.9%).

Table 4 shows the results of univariable logistic regression analysis testing associations with “perceived need for information”. These analyses revealed that only women with a more positive birth experience had lower odds of “perceived need for information” (OR 0.86 95% CI 0.79–0.93). The remaining associations were not significantly associated with “perceived need for information”.

**Table 4:** Univariable and multivariable logistic regression analysis of perceived need for information

	Total N	Need for information n (%)	Crude OR (95% CI)	Adjusted OR (95% CI)	*p*- value
Total study population	594	273 (46%)			
Maternal characteristics					
Parity					
Primiparous	328	150 (45.7)	reference	-	
Multiparous	266	123 (46.2)	1.02 (0.74-1.41)	-	
Maternal age					
<30	239	107 (44.8%)	0.91 (0.64-1.31)	-	
30-34	238	112 (47.1%)	reference	-	
≥35	117	54 (46.2)	0.96 (0.62-1.50)	-	
Gestational age					
<37 weeks	28	14 (50)	1.24 (0.58-2.66)	-	
37-40 weeks	443	198 (44.7)	reference	-	
≥ 41 weeks	123	61 (49.6)	1.22 (0.82-1.82)	-	
Ethnic background					
Dutch	510	229 (44.9)	reference	-	
Western	59	32 (54.2)	1.45 (0.95-2.50)		
Non-Western	24	12 (50)	1.23 (0.54-2.78)	-	
Education level					
Low	43	19 (44.2)	0.87 (0.46-1.65)	-	
Middle	207	90 (43.5)	0.84 (0.60-1.19)	-	
High	344	164 (47.7)	reference	-	
Body Mass Index (imputed)					
< 18 kg/m^2^	10	5 (50)	1.10 (0.31-3.78)	-	
18.5 – 24.9 kg/m^2^	246	117 (47.56)	reference	-	
25 – 29.9 kg/m^2^	192	82 (42.7)	0.83 (0.56-1.23)	-	
≥ 30 kg/m^2^	140	66 (47.1)	1.00 (0.66-1.53)	-	
Negative sexual experience					
Yes	174	90 (51.7)	1.39 (0.97-1.98)	1.33 (0.93-1.90)	0.12
No	420	183 (43.6)	reference	reference	
Postpartum at					
0-5 months	90	37 (41.1)	reference	-	
6-11 months	228	112 (49.1)	1.38 (0.84-2.27)	-	
12-17 months	152	64 (42.1)	1.04 (0.61-1.77)	-	
18+ months	121	58 (47.9)	1.32 (0.76-2.29)	-	
Obstetric and medical factors					
Mode of conception					
Spontaneous	539	250 (46.4)	reference	-	
Non-spontaneous	53	22 (41.5)	0.82 (0.46-1.45)	-	
Mode of Birth					
Spontaneous Vaginal	462	201 (43.5)	reference	-	
Assisted vaginal birth	52	29 (55.8)	1.64 (0.92-2.92)	-	
Caesarean Section	80	43 (53.8)	1.51 (0.94-2.43)	-	
Episiotomy					
Yes	120	60 (50.0)	1.23 (0.82-1.83)	-	
No	474	213 (44.9)	reference	-	
Perineal trauma that needed stitching					
Yes	178	84 (47.2)	1.07 (0.76-1.53)	-	
No	416	189 (45.4)	reference	-	
Breastfeeding					
No	109	47 (43.1)	reference	-	
Yes, 0-3 months	150	68 (45.3)	1.09 (0.67-1.80)	-	
Yes, 3-6 months	66	28 (42.4)	0.97 (0.52-1.80)	-	
Yes, > 6 months	269	130 (48.3)	1.23 (0.79-1.93)	-	
Birth experience, psychological factors and relationship assessment					
Birth experience Score from 1 to 10			**0.86 (0.79-0.93)**	**0.86 (0.79-0.94)**	**<0.001**
Psychological complaints					
Yes	67	38 (56.7)	1.63 (0.98-2.72)	-	
No	527	235 (44.6%)	reference	-	
Relationship assessment scale from 1 to 7			0.91 (0.69-1.19)	-	

After performing the different steps of the planned multivariable hierarchical logistic regression, positive birth experience was again the only factor that was associated with lower odds of “perceived need for information” (OR 0.86, CI 0.79 to 0.94).

## Discussion

### Key findings

The current survey among postpartum women in the Netherlands shows that approximately half of the participating women experienced postpartum sexual health problems. Sexual distress and sexual dysfunction were more common in women with a negative sexual experience, and less common when a higher level of relationship satisfaction was reported. Perineal trauma requiring stitching is associated with increased odds of sexual dysfunction. Obesity was associated with decreased odds of sexual dysfunction. In addition, a positive childbirth experience was associated with decreased odds of sexual distress and decreased odds of “perceived need for information”. Approximately half of the women felt the need for (more) information about sexual health postpartum. A quarter of all women reported that they actually discussed sexual health with their midwives.

### Comparison with other studies and interpretation of the findings

Remarkably, this study revealed that Dutch postpartum women, i.e., half of the participants in the current survey, experienced sexual health problems postpartum (43.7% sexual dysfunction, 52.3% distress). The rate of sexual dysfunction in our study was lower than that reported in an earlier Australian study (64.3–70.5%), but almost identical to that reported in a previous Irish study, 40-50%^5,7^. A possible reason for this difference from the Australian study is that they looked at dysfunction in the first year postpartum and our study included women up to 24 months postpartum [5]. Another reason for the distinct outcomes could be that there was a 10-year interval between the data-collection of our study and that of the Australian study. In the meantime, women might feel more comfortable discussing sexual health issues with their prenatal care provider since it is more ‘normal’ than it was 8 years ago, which might lower their degree of sexual health problems. The difference in sexual health problems in postpartum women can also be related to the fact that the Netherlands is known for it’s favourable sexual health outcomes due to various socio cultural factors [17], which could also be the case in Dutch postpartum women. The high rate of women experiencing sexual distress is concerning, but we found no previous studies to compare this with. However, among 203 primiparous Canadian women, sexual distress was reported to increase from pregnancy until 3 months postpartum, and then to decrease [32].

### Sexual dysfunction and distress

Other studies have shown that women with negative or non-consensual sexual experiences have poorer sexual functioning [33] In our study, this association was shown in postpartum women. Mechanisms that might contribute to sexual dysfunction in women with a negative sexual experience are the use of avoidance behaviours (either by abstinence of sexual activities or the use of distraction, dissociation or the use of substances to alter awareness during sexual activities), which might prevent the experience of potentially corrective sexual experiences [34]. In women with a history of sexual abuse in childhood/ adolescence, avoidance tendencies were shown to explain lower orgasm function [34]. In addition, childbirth might trigger negative memories and reliving of negative sexual experiences [35]. It can be hypothesised that this could negatively influence sexual health in the postpartum period. Maternity care staff should demonstrate respect and support women in remaining in control and in feeling safe during labour, so that the reenactment of abuse is minimised [35].

In contrast to earlier studies we did not observe that primiparity and breastfeeding were associated factors for sexual health problems [5, 7, 9]. These differences in findings between these earlier studies and the current study could be explained by the use of different measurement instruments to assess sexual health. For example, O’Malley [7] and Handelzalts [10] did not use the FSFI or FSDS to assess sexual health. Moreover, in the study of O’Malley et al. [7], breastfeeding was associated with sexual health problems when they were breastfeeding until 6 months postpartum, but not at 1 year postpartum. In our study half of the participants had given birth more than one year prior.

Furthermore, we found that better relationship satisfaction is associated with fewer sexual health problems. This finding is in accordance with that of Khajehei et al. [5], who reported that sexual function was negatively associated with relationship dissatisfaction in women within 12 months postpartum. However, it is impossible, owing to the cross sectional design to determine which factor is the cause and which factor is the effect of these sexual and relationship problems. Studies have shown that the associations of sexual health with well-being and with relationship quality are bidirectional across time [36, 37].

We also observed that perineal trauma that needed stitching was associated with increased odds of sexual dysfunction, which is in line with previous research. For example, Rathfish et al. [38] reported that, compared with women with intact perineum, those who had second degree tears had lower levels of libido, orgasm and sexual satisfaction. O’Malley et al. [7] reported that second- and third-degree tearing were associated with dyspareunia six months postpartum. Limiting perineal trauma during childbirth is therefore important for sexual function postpartum. However, perineal trauma is not always preventable, for this reason, preparing women and their partners during the antenatal period, by providing information about this potential complication, may reduce stress and anxiety about resuming intercourse and possibly reduce problems [7].

Mode of birth was not associated with sexual health problems in our study. In contrast, a recent systematic review [12] reported this association. However, many studies included in the review showed no association between mode of birth and postpartum sexual functioning and an earlier review also did not find an effect of mode of birth and sexual function [39].

Being obese seems to decrease the odds of experiencing sexual health problems postpartum. O’Malley et al. [7] reported that obese postpartum women were less likely to experience a lack of vaginal lubrication at 6 months postpartum, which confirms our findings. However, in the general population, obesity is associated with increased odds of sexual dysfunction [40]. Further research is necessary to further explain these findings.

In our study, women with positive birth experiences were less likely to experience sexual distress and had lower odds of “perceived need for information” about sexual health. A negative childbirth experience can have direct and long-term negative effects [41]. Handelzats [10] stated: “It appears that the psychological aspects of mode of birth (childbirth experience) may be what influences both sexual functioning and sexual satisfaction, rather than the more technical physical aspects of mode of birth.” Furthermore, Handelzats [10] reported that childbirth experience is very important for many aspects of postpartum wellbeing. A traumatic experience and the feeling of losing control, especially in a life-changing event such as childbirth, could have an impact on various aspects of one’s life. Our study specifically shows that sexual wellbeing is one of these aspects. One might hypothesise that a positive mental health state due to an empowering birth experience could positively influence sexual wellbeing.

### Perceived need for information

To our knowledge this is the first study reporting that women who have a positive birth experience have less “perceived need for information”. The previous study among Canadian women revealed that 76% of women felt the need to discuss sexual activity with their caregiver [13]. In this study 46.0% of the women wished for more information. A possible explanation for these differences is that the previous study collected data about the need for information in sexual health during pregnancy, whereas our study assessed the “perceived need for information” in the postpartum period. In addition, the previous study was performed more than 20 years earlier, possibly there have been changes over time [13]. However, in our study, only a quarter of the women received information about sexual changes. This indicates that it is not a routine topic to discuss in maternity care.

## Strengths and limitations

This study has strengths and limitations that need to be discussed.

This is the first study that uses both the FSFI and FSDS questionnaires to assess sexual health in Dutch postpartum women. Both are validated questionnaires measuring two important and distinct aspects of sexual health, i.e. sexual function and sexual distress. Additionally, to our knowledge, the RAS questionnaire has not been used before in combination with the FSFI and FSDS among postpartum women in a Dutch setting. This provides better insight into the interconnectedness between partner relationship satisfaction and sexual health.

A limitation is that, in contrast to the longitudinal study by O’Malley et al. [7], we were not able to assess and illustrate a change in sexual problems across the postpartum period due to the cross-sectional design of the current survey. Among the women in our study, there were differences in time since birth when completing the questionnaire.

Some of the questions in the questionnaire addressed past experiences, such as mode of birth. Recollection of memories can lead to recall bias. Furthermore, it might be more difficult to define factors when asking women, such as perineal trauma that needed stitching, which possibly includes vaginal or labium ruptures that needed stitching as well. It may have been more reliable if medical files were used to collect this information. It is possible that women with sexual problems may have a better recollection of trauma, in which case the way we asked about perineal trauma may have introduced bias. However, Ye et al. [42] found that patient reporting provides similar information to medical records and the association we found between sexual dysfunction and perineal trauma, is also in line with findings from other studies [7, 38], reducing the risk of bias.

Selection bias could have been caused by the way the questionnaire was distributed during the recruitment period. The questionnaire was posted on different social media platforms such as Facebook, Linked-in and Instagram. Only women who used or followed these groups or pages would have come across the questionnaire. Groups and influencers post about a certain topic, so only women interested in these topics would come across the questionnaire, possibly attracting a selective audience. It seems unlikely that the associations shown in our study would be in the opposite direction in the full population.

Our sample differed in some ways in demographic characteristics compared with the Dutch postpartum population. This study included 8.5% low educated women, 35.3% middle educated women and 56.3% high educated women compared with 28.5%, 36.0% and 33.4% respectively in the Netherlands [26]. For ethnic background, 10.6% of our sample had a Western migration background which is similar to the 10.5% of the Dutch population. However, only 3.7% of our population had a non-Western migration background, whereas 13.7% of the Dutch population did. It is unlikely that the differences in population caused a difference in results, because most of the associated factors for sexual health in our study were similar to those reported in other studies. Nevertheless, as the current sample was healthier than the average Dutch female population, the prevalence of sexual health problems might have been underestimated. Additionally, one of our inclusion criteria was understanding the Dutch language. However, we did not ask whether women gave birth in the Netherlands. Thus, we cannot completely rule out that a small number of subjects in our sample may have given birth outside the Netherlands and were not fully representative of the Dutch system.

The ‘changes in sexual health’ item may have some limitations especially without a clear recall period or reference point. For example, some women may have responded in reference to recall of initial changes; and others to more recent changes. If women were reporting on their current experiences, this may not have captured changes that resolved or changed. This may make it difficult to draw conclusions about the exact timing of the results based on this measurement.

The perceived need for information questions were not filled in by 47 of 641 participants, which might have biased our results. The reason for noncompletion may have been due to the time it took the postpartum women to fill in all items on the questionnaire, and as the questions with regard to “perceived need for information” were placed at the end of the questionnaire, some women may have stopped completing it, due to time constraints. It may be that women who completed the questionnaire were more likely to “perceive need for information” and therefore the prevalence of the latter may be slightly overestimated.

Last, a limitation of our study is that the FSFI questionnaire has not been developed or validated for postpartum women [43]. Postpartum sexuality is very complex, including major changes in a woman’s life and relationship [43]. This may mean that classifying women as sexually dysfunctional based on FSFI may be less accurate [43]. However, this is in line with other studies who also used FSFI in a postpartum population [5, 44]. Future research is needed to adapt and validate FSFI for postpartum women to improve its accuracy for this population.

## Recommendations

A total of 24.2% of the women discussed their sexual health postpartum with their midwives during pregnancy or the postpartum period. However, 46.0% of the total sample wanted this subject to be discussed, suggesting only a proportion of women are getting their needs met. For this reason, we recommend that maternity care providers discuss this topic with their clients on a routine basis. In particular midwives, because 82.2% of women reported that they wanted to discuss sexual health with their midwife. Most women want this subject to be discussed at the 6 week check-up or between 6 weeks and 3 months postpartum. This may be because sexual life begins again during this period; it was reported that more than half of the women resumed sexual intercourse by 3 months postpartum [45]. Since women have a check-up at 6 weeks postpartum, this is a good moment to discuss this subject.

In our study women with a positive childbirth experience had decreased odds of having sexual health problems and a lower “perceived need for information” concerning discussing sexual health postpartum. Maternity care providers should therefore aim to support and facilitate women in having positive birth experiences as much as possible. Effective strategies to promote a positive birth experience include supporting women during birth, intrapartum care with minimal interventions and birth preparedness [46].

Women with negative sexual experiences have more sexual health problems. However, these women did not report a greater “perceived need for information”. A possibility should be given to these women to openly talk about their sexual health and their past experiences. Approximately half of women with a negative sexual experience confide in their provider [47]. Women reported that “the right environment” for disclosure of such an experience constitutes an important condition [47]. In addition, care providers should be alert to unspoken messages and employ ‘universal precautions’ to mitigate hidden trauma [35].

Women with perineal trauma that needed stitching experience more sexual dysfunction. Trying to preserve the perineum as much as possible in the second stage of labour is important.

Previous studies have shown that healthcare professionals lack competence and confidence in their ability to help with sexual health problems [7]. A method that could be used is the PLISSIT-model, a model for discussing sexual health with clients which could therefore help healthcare professionals to address this matter. This model has been developed to help healthcare workers in different fields to communicate with clients about sexual health and changed sexual health after life-changing (health) events and support them with questions about sexual health [48]. Prenatally discussing physical, emotional, sexual and lifestyle changes that often occur postpartum with a maternity care provider can promote postpartum sexual satisfaction [3]. Maternity care providers can play an important role in discussing sexual health and possible pre-existing sexual health problems, because they see pregnant women and their partners often during pregnancy [9]. After all, all care providers, including maternity care providers, bear responsibility for the overall health of their clients, which includes sexual health [49].

As important as it is to prepare women for the challenges of postpartum sexual health we would advise referring women or couples for appropriate care, if significant problems with sexuality do arise. Referral to specialised care such as psychological treatment may be appropriate, however, little to no research has been done to evaluate treatment options for sexual problems in postpartum women [50]. One option is to refer couples to the #postpartumhankypanky videos [51]. These are videos that inform and educate couples and professionals about what is known about postpartum sexual health [51].

## Conclusion

Approximately half of postpartum women in the Netherlands experience sexual health problems. Associated factors for sexual health problems (dysfunction and/or distress) were a history of negative sexual experience and perineal damage. Obesity, positive birth experience and relationship satisfaction have been shown to be protective for sexual health problems.

Midwives should provide all pregnant and postpartum women with adequate information about their sexual health postpartum, with special attention given to the childbirth experience. Most women wanted to have sexual health discussed six weeks to three months postpartum by their midwives and the check-up at six weeks postpartum is a good moment to discuss this matter. Special attention should also be given to women with negative sexual experiences even though they do not show more “perceived need for information”, but they do have more sexual health problems.

## Supplementary Information

Supplementary Material 1.

## Data Availability

The datasets generated and/or analysed during the current study are not publicly available due to guarantee the privacy of the participants, but are available from the corresponding author on reasonable request.

## Abbreviations

adjOR: adjusted Odds Ratio
AVAG: Academie Verloskunde Amsterdam Groningen
BMI: Body Mass Index
CI: Confidence Interval
FSDS: Female Sexual Distress Scale
FSFI: Female Sexual Function Index
KNOV: Koninklijke Nederlanse Organisatie voor Verloskundigen
METC: Medisch Ethische Toetsings Commissie
NVOG: Nederlandse Vereniging Obstetrie en Gynaecologie
OR: Odds Ratio
PLISSIT: Permission Limited Information Specific Suggestions Intensive Therapy
RAS: Relationship Assessment Scale
SPSS: Statistical Package for Social Sciences
VU: Vrije Universiteit
