# Supplementary material for: Sexual health after childbirth in Dutch women: prevalence, associated factors and perceived need for information: a cross-sectional study
Source: BMC Pregnancy Childbirth. 2024 Dec 20;24:838. doi: 10.1186/s12884-024-06918-w (PMC11660435; doi:10.1186/s12884-024-06918-w)
Supplement: Supplementary file 1 — Supplementary Material 1. [file 12884_2024_6918_MOESM1_ESM.pdf]

Supplementary table 1. Presenting p-values of the variables that were included in the separate steps in the multivariable logistic hierarchical model regression analysis with sexual dysfunction, sexual distress and “perceived need for information” as the outcome variables.

	**Sexual dys-function**				**Sexual distress**				**Need for care**		
	Step 1	Step 2	Step 3		Step 1	Step 2	Step 3		Step 1	Step 2	Step 3
Maternal characteristics											
**Parity**											
Primiparous	reference				reference				reference		
Multiparous	0.19				0.54				0.91		
**Maternal age**											
<30	0.17				0.98				0.69		
30-34	reference				reference				reference		
≥35	0.71				0.37				0.86		
**Gestational age**											
<37 weeks	0.89				0.49				0.69		
37-40 weeks	reference				reference				reference		
≥41 weeks	0.29				0.65				0.35		
**Ethnic background**											
Dutch	reference				reference				reference		
Western	0.60				0.89				0.17		
Non-Western	0.75				0.34				0.75		
**Education level**											
Low	0.23				0.96				0.55		
Middle	0.53				0.69				0.29		
High	reference				reference				reference		
**Body Mass Index (imputed)**											
18,5-24.9 kg/m^2	reference	reference	reference		reference	reference			reference		
25-29.9 kg/m^2	0.23	0.37	0.15		0.96	0.72			0.52		
≥30 kg/m^2	**0.003**	**0.02**	**0.001**		0.09**	0.17			0.74		
**Negative sexual experience**											
Yes	**0.01**	**0.02**	**0.04**		**<0.001**	**<0.001**	**0.01**		0.10**	0.07**	0.12**
No	reference	reference	reference		reference	reference	reference		reference	reference	reference
**Postpartum at**											
0-5 months	reference	reference			reference				reference		
6-11 months	0.28	0.36			0.29				0.19		
12-17 months	0.14**	0.16			0.96				0.85		
18+ months	0.20	0.23			0.30				0.29		
Obsteric and medical factors											

**Mode of conception**											
Spontaneous		reference				reference				reference	
Non-spontaneous		0.59				0.90				0.48	
**Mode of birth**											
Spontaneous vaginal		reference				reference				reference	reference
Assisted vaginal birth		0.89				0.50				0.16	0.24
Caesarean Section		0.98				0.39				**0.05**	0.31
**Episiotomy**											
Yes		0.95				0.30				0.57	
No		reference				reference				reference	
**Perineal trauma that needed stitching**										0.28	
Yes		**0.04**	**0.03**			0.61					
No		reference	reference			reference				reference	
**Breastfeeding**											
No		reference				reference				reference	
Yes, 0-3 months		0.55				0.97				0.76	
Yes, 3-6 months		0.62				0.40				0.93	
Yes, more than 6 months		0.23				0.73				0.34	
Birth experience, psychological factors and relationship assessment											
**Birth experience Score from 1 to 10**			0.30				**0.01**				**0.01**
**Psychological complaints**											
Yes			0.90				0.45				0.30
No			reference				reference				reference
**Relationship assessment scale from 1 to 7**			**<0.001**				**<0.001**				0.71

p-values <0.05 are indicated in bold; these variables are included in the following step.

**p-value <0.15; they are included in the following step.
